# The Portrait of Adolph Lippe, M. D.

**Published:** 1892-04

**Authors:** 


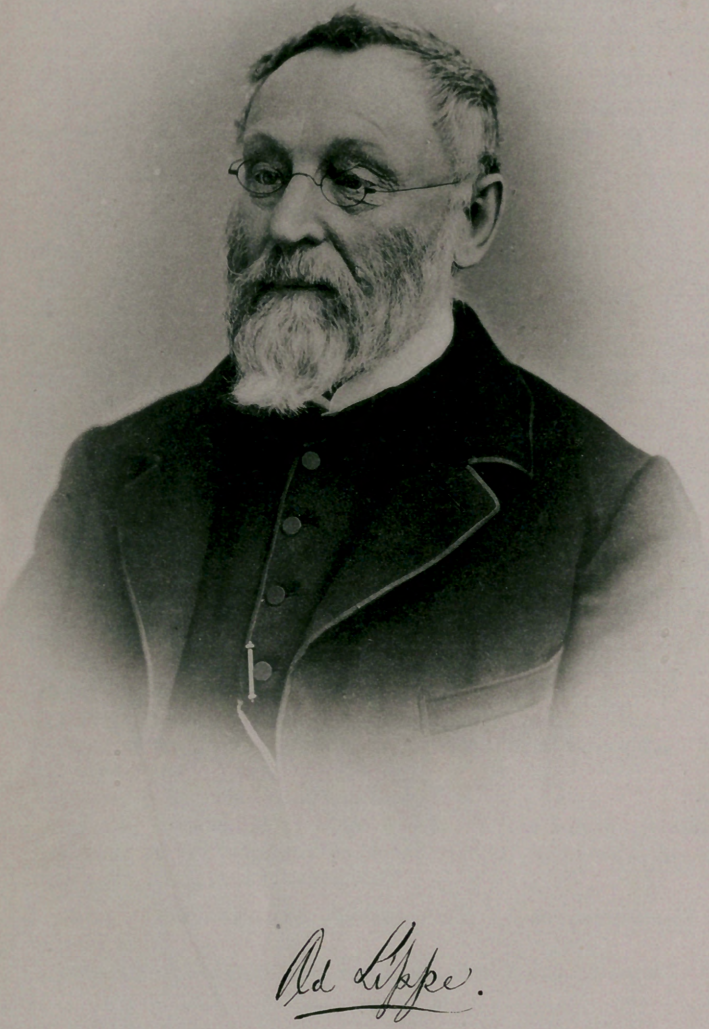

*The Homeopathic Physician,*
*April 1892.*

*Copyright 1892.*

THE

### HOMŒOPATHIC PHYSICIAN,

A MONTHLY JOURNAL OF

HOMŒOPATHIC MATERIA MEDICA AND CLINICAL MEDICINE.

"If our school ever gives up the strict inductive method of Hahnemann, we are lost, and deserve only to be mentioned as a caricature in the history of medicine."—CONSTANTINE HERING.

Vol. XII. APRIL, 1892. No. 4.

### EDITORIAL.

THE PORTRAIT OF ADOLPH LIPPE, M. D.—The frontispiece which graces this number of THE HOMŒOPATHIC PHYSICIAN will be at once recognized by all homœopathists as a most vivid portrait of the late Dr. Lippe, so well and so favorably known as the foremost homœopathic physician in Philadelphia.

So many requests for a copy of the venerable Hahnemannian's photograph have been made of the editor that it has been determined that these wishes shall be granted. Accordingly the accompanying picture is now offered to the profession not alone as a gratification to the subscribers, but as a modest memorial on the part of this journal to our beloved friend and master.

Shortly after Dr. Lippe's death, certain of his professed friends proposed a memorial to his honored name. A committee was formed and subscriptions were procured for this most worthy object. The memorial was to take the shape of a series of lectures by distinguished homœopathic physicians upon subjects relating to Homœopathy. A sufficient amount was stated to have been raised, according to the reports of the committee. This project was considered more worthy of the illustrious scholar who through his whole life had been the most persistent, logical, and able defender of the great law of the similars than would be any brass or stone tablet that could be erected to his memory in either church or hospital. Invitations were issued to a number of the prominent homœopathists to deliver the lectures of this memorial, and thus the plan was started with great enthusiasm. But whether the amount of capital raised was found to be insufficient, or *what* was the cause of failure, certain it is that this well-devised scheme turned into vapor and vanished in oblivion.

Therefore Dr. Lippe yet remains without a fitting memorial.

As THE HOMŒOPATHIC PHYSICIAN was the journal which was founded by Dr. Lippe, and the one to which he was deeply attached, and the one whose success engaged his most ardent solicitude, it seems to be the most fitting vehicle in which to convey to his true and earnest followers a memorial that will be at once gratifying and lasting.

The negative from which this picture is printed was taken twenty-four years ago. After much search it was found, and the consent of Dr. Lippe's surviving son having been obtained, the order was given and the accompanying remarkably fine portrait was executed, at considerable trouble and expense, and is now given to the profession.

Dr. Lippe's personal appearance had not sensibly changed since this portrait was taken, consequently it represents him exactly as he appeared a few days before his death.

Our readers, we are well aware, will appreciate the work we have done, and hail with pleasure the opportunity to procure so elegant a souvenir of the departed apostle of Hahnemann.

